# Transduction and Adaptation Mechanisms in the Cilium or Microvilli of Photoreceptors and Olfactory Receptors From Insects to Humans

**DOI:** 10.3389/fncel.2021.662453

**Published:** 2021-04-01

**Authors:** Fatima Abbas, Frans Vinberg

**Affiliations:** Vinberg Lab, Department of Ophthalmology and Visual Science, John A. Moran Center, University of Utah, Salt Lake City, UT, United States

**Keywords:** adaptation, olfaction, activation, inactivation, phototransduction cascade

## Abstract

Sensing changes in the environment is crucial for survival. Animals from invertebrates to vertebrates use both visual and olfactory stimuli to direct survival behaviors including identification of food sources, finding mates, and predator avoidance. In primary sensory neurons there are signal transduction mechanisms that convert chemical or light signals into an electrical response through ligand binding or photoactivation of a receptor, that can be propagated to the olfactory and visual centers of the brain to create a perception of the odor and visual landscapes surrounding us. The fundamental principles of olfactory and phototransduction pathways within vertebrates are somewhat analogous. Signal transduction in both systems takes place in the ciliary sub-compartments of the sensory cells and relies upon the activation of G protein-coupled receptors (GPCRs) to close cyclic nucleotide-gated (CNG) cation channels in photoreceptors to produce a hyperpolarization of the cell, or in olfactory sensory neurons open CNG channels to produce a depolarization. However, while invertebrate phototransduction also involves GPCRs, invertebrate photoreceptors can be either ciliary and/or microvillar with hyperpolarizing and depolarizing responses to light, respectively. Moreover, olfactory transduction in invertebrates may be a mixture of metabotropic G protein and ionotropic signaling pathways. This review will highlight differences of the visual and olfactory transduction mechanisms between vertebrates and invertebrates, focusing on the implications to the gain of the transduction processes, and how they are modulated to allow detection of small changes in odor concentration and light intensity over a wide range of background stimulus levels.

## Introduction

Phototransduction and olfaction have been widely investigated in both invertebrate (Hardie and Raghu, 2001; Touhara and Vosshall, 2009; Yau and Hardie, 2009; Hardie and Juusola, 2015; Honkanen et al., 2017; Fleischer et al., 2018; Schmidt and Benton, 2020) and vertebrate (Schild and Restrepo, 1998; Burns and Baylor, 2001; Fain et al., 2001; Arshavsky et al., 2002; Luo et al., 2008; Kaupp, 2010; Pifferi et al., 2010; Vinberg et al., 2018) species. Signal transduction in vertebrate olfactory sensory neurons (OSNs) and photoreceptors (PRs) both rely upon G protein signaling cascades, while using distinct classes of G and effector proteins: A G-protein-coupled receptor (GPCR) detects a stimulus and modulates the open probability of cyclic nucleotide-gated (CNG) ion channels *via* classical GPCR signaling cascades involving the activation of an effector enzyme (E), adenylyl cyclase (AC) in the OSN and phosphodiesterase (PDE) in photoreceptors. This leads to a change in the membrane potential (V_m_) of the primary sensory neuron that can be propagated to the sensory centers of the brain. Invertebrates appear to use a wider range of signaling cascades to detect odors or light. Their phototransduction is mostly mediated by a G_q_-coupled pathway controlling transient receptor potential (TRP) and transient receptor potential like (TRPL) channels in the plasma membrane of the microvillar compartments of the photoreceptor neuron. However, there are some invertebrate species that also utilize the release of Ca^2+^ from intracellular endoplasmic reticulum (ER) stores *via* inositol triphosphate (IP_3_) pathway, or the same phototransduction pathway found in vertebrates. Some details of invertebrate phototransduction are unknown, most notably the second messenger gating their transduction channels remains to be resolved. Olfactory signal transduction in invertebrates is not understood in detail, however, it is often mediated by ligand-gated odor receptor (OR)/co-receptor (Orco) heteromer that may also be regulated by metabotropic pathway(s) (Wicher et al., 2008; Nakagawa and Vosshall, 2009; Hansson et al., 2010). In addition, some odors are detected *via* a purely ionotropic receptors (IRs) reminiscent of ionotropic glutamate receptors widely used in chemical synaptic transmission between neurons. In this review we will describe visual and olfactory transduction in vertebrates and invertebrates, focusing on how their differences contribute to the gain of the visual and odor transduction in vertebrates and invertebrates as well as how this gain is modulated to allow adaptation of vision and olfaction over a wide range of background light and odorant levels.

## Transduction Cascades In the Primary Sensory Neurons of Olfactory and Visual Systems

**Figure 1:**
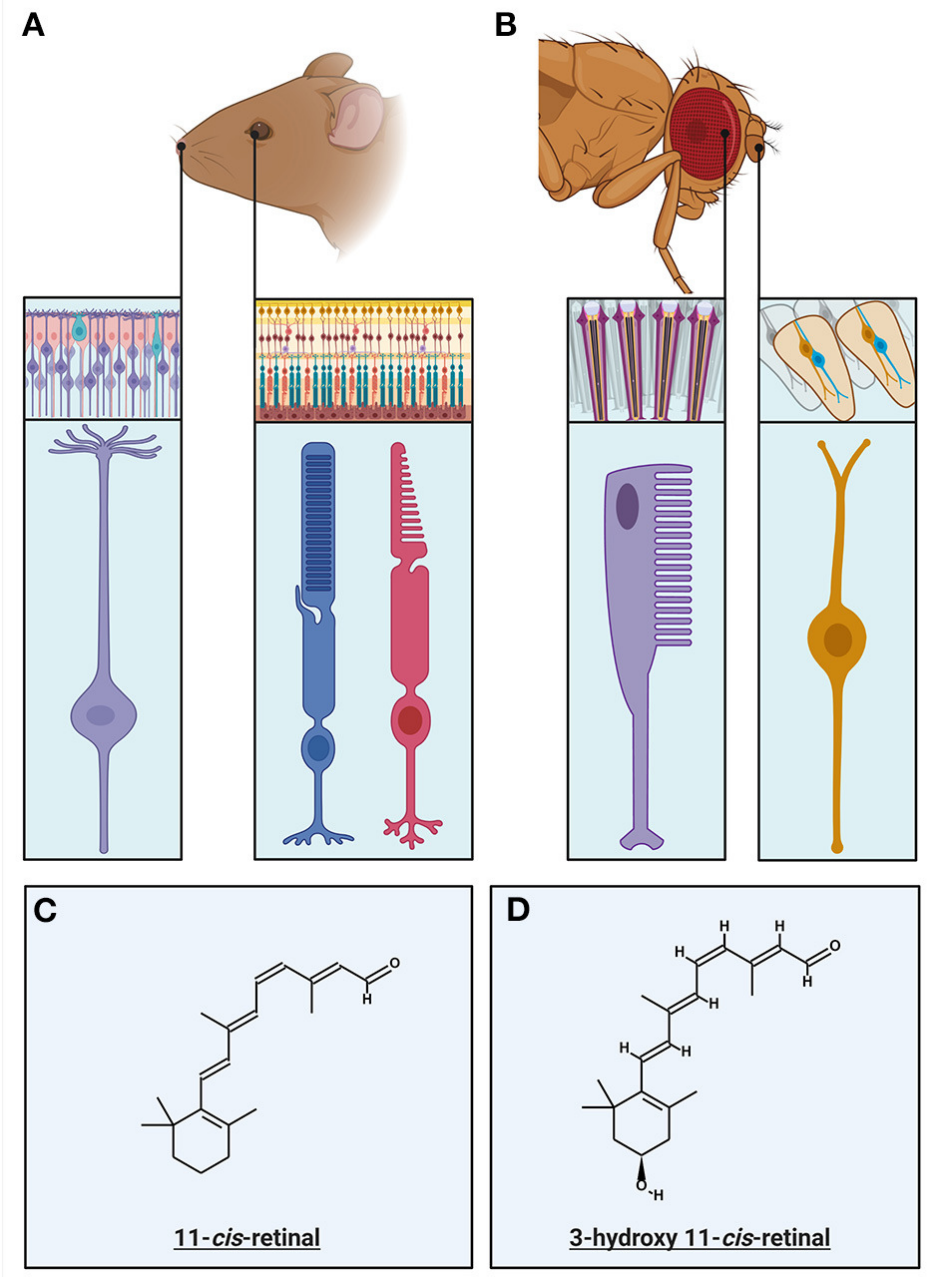
Primary Sensory Neurons of the invertebrate and vertebrate visual and olfactory sensory systems: **(A)** The primary sensory neurons in the vertebrate olfactory and visual systems and their respective circuitry. Rods (blue) and cones (magenta) mediate vision at different light intensities. Rods mediate low light vision and having specializations enabling this, detailed within this review. Cones mediate daylight color vision and have specific specializations that mediate this. The olfactory sensory neurons (purple) have multiple cilia protruding from the end of the neuron into the olfactory lumen, in which the olfactory transduction machinery, including olfactory receptors, are localized. **(B)** The primary sensory neurons in the invertebrate (*Drosophila*) olfactory and visual systems, within their respective circuitry. The invertebrate ommatidium is the structure in which invertebrate photoreceptors (rhabdomeres) are coupled with the support and pigment cells. The rhabdomere (purple) is primary sensory neuron in which the visual transduction machinery is localized within the villi. The olfactory sensory neurons (OSNs) are located within sensory hairs, sensilla. OSNs (orange) project dendrites into the sensillum, with the olfactory receptors expressed on the surface. **(C)** The chemical structure of the chromophore responsible for vertebrate visual transduction, 11-*cis*-retinal. **(D)** The chemical structure of the chromophore present in Microvillar photoreceptors, 3-hydroxy-11-*cis*-retinal.

Specialized light-detecting neurons are present in nearly all animals and can be classified into two types: ciliary and microvillar PRs (Fain et al., 2010). Ciliary photoreceptors are formed through membrane invaginations in the outer segment, resulting in compact disk-like structures (Figure 1A, inset). These disks create a large surface area with the membranes containing a high concentration of visual pigments and phototransduction cascade proteins (Sjöstrand and Kreman, 1978; Gilliam et al., 2012). Rhabdomeric photoreceptors form microvillar evaginations containing the photopigment and phototransduction machinery (Figure 1B, inset). Almost all vertebrates use ciliary PRs for their image-forming vision whereas microvillar PRs are found in most invertebrates. However, some invertebrate species, such as box jellyfish or scallops, also have ciliary PRs (Arendt et al., 2004; Fain et al., 2010).

In mammals, the primary sensory neurons of the olfactory system are clustered in the olfactory epithelium at the back of the nasal cavity (Buck and Axel, 1991; Mombaerts, 1999). These are bipolar neurons, with an axon that extends to the olfactory bulb, and a dendrite with several cilia which extend into the olfactory lumen (Figure 1A, inset; Menco, 1997). These cilia are the location of the olfactory receptors (ORs) as well as the concentrated localization of the components of olfactory transduction. The vomeronasal organ (VNO) is located at the base of the nasal septum, and is primarily thought to be involved in sensing of pheromones (for review see Dulac and Torello, 2003). Since its' neurons do not project to the main olfactory bulb and contain vomeronasal receptors that are distinct to the olfactory receptors, the VNO is considered part of the Accessory Olfactory System (AOS). Interestingly there is evidence that the vomeronasal system is no longer functional in primates (Meredith, 2001; Zhang and Webb, 2003). For simplicity, this review will primarily focus on transduction in olfactory receptor neurons projecting to the main olfactory bulb. In insects, the OSNs are located on sensory hairs called sensilla on the antennae and maxillary palp (Figure 1B, inset; Pellegrino and Nakagawa, 2009).

In contrast to the graded potentials transmitted by most PRs, both vertebrate and invertebrate OSNs fire action potentials in response to the depolarization caused by the activation of olfactory transduction in the cilia. However, there are some invertebrates with photoreceptor axons projecting directly to the visual centers of the brain, generating regenerative action potentials to enable signal transmission without attenuation (Hartline, 1938). This review will focus on the photoreceptors that do not generate action potentials.

Below, we will describe transduction activation and inactivation as well as adaptation mechanisms in PRs and OSNs of vertebrates and invertebrates. In addition, we will compare the contribution of these mechanisms to the gain of transduction cascades and how that gain is modulated *via* various feedback mechanisms to enable detection of changes in odorant concentration or light intensity over a wide range of background stimulus levels.

## Vertebrate Transduction Cascades

**Figure 2:**
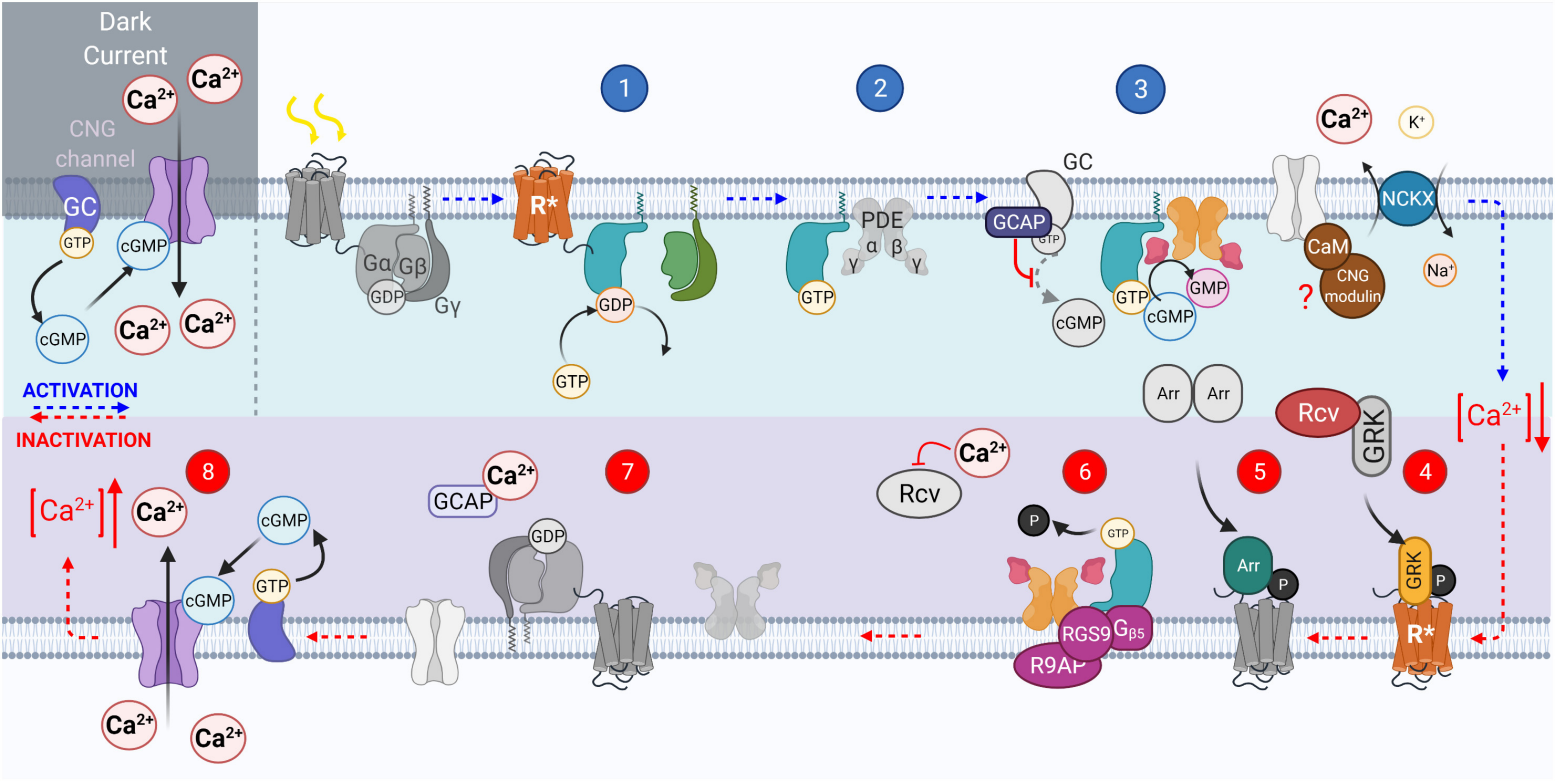
The vertebrate phototransduction cascade, inactivation, and adaptation mechanisms. The key steps within the vertebrate phototransduction cascade (blue numbering from top left to right) and its subsequent inactivation (red numbering from bottom right to left). In the dark, the CNG channel maintains a dark current, in which Ca^2+^ ions influx through open CNG channels as the Guanyl Cyclase enzyme is constituently active. Upon absorption of a photon, the chromophore 11-*cis*-retinal is isomerized to all-*trans*-retinal, causing the receptor to become activated (R*). In turn, the associated G protein, transducin, is activated with the exchange of GDP for GTP, ${\text{G}}_{\text{T}}\alpha^{*}$ (step 1). ${\text{G}}_{\text{T}}\alpha^{*}$ then displaces PDEβγ subunits (step 2). The displaced PDEγ subunit allows cGMP to be reduced to GMP, reducing the concentration of cGMP, closing CNG channels, causing a hyperpolarization of the photoreceptor (step 3). The activated receptor, R*, is inactivated by GRK mediated phosphorylation (step 4). Followed by Arrestin binding (step 5). The GAP protein complex formed of RGS9, R9AP, and Gβ5 bound to both PDEγ and activated ${\text{G}}_{\text{T}}\alpha^{*}$ causes hydrolysis of GTP on the G_T_α to GDP (step 6). G_T_α then dissociates from PDE, reassociating to the βγ subunits, and PDEγ also inhibits the PDEαβ to prevent cGMP hydrolysis (step 7). Intracellular Ca^2+^ concentration is raised as CNG channels reopen in response to the increasing cGMP concentration, reestablishing the dark current (step 8).

**Figure 3:**
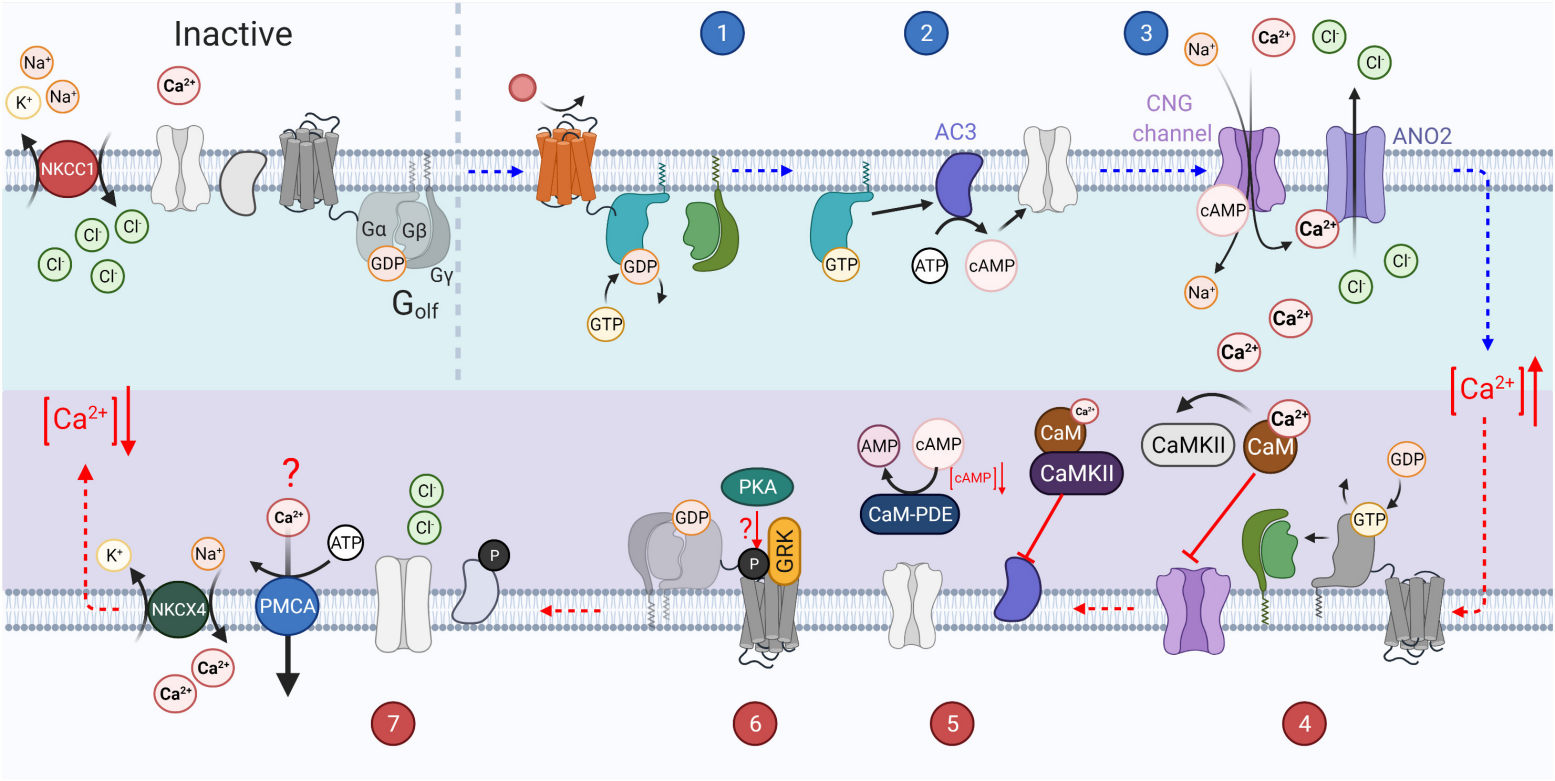
The vertebrate olfactory transduction cascade, inactivation, and adaptation mechanisms: OSNs maintain a high intracellular Cl^−^ ion concentration through the constitutive activity of the NKCC1 transporter, which transports Cl^−^ ions into the cell using the energy from the export of Na^+^ and K^+^ ions. An odorant binds to the G-protein coupled olfactory receptor (OR) on the cilium of the sensory neuron. The activated receptor in turn activates G_olf_ (step 1). The Gα_olf_ subunits then activate adenylyl cyclase III (AC3) which catalyzes the conversion of ATP to cAMP (step 2). The increasing concentration of cAMP causes the opening of CNG channels on the membrane. This causes an influx of Ca^2+^ ions. The increasing Ca^2+^ concentration also causes the opening of the calcium activated chloride ion channel, ANO2, which further depolarizes the cell as the increased intracellular chloride gradient causes an efflux of Cl^−^. The increased Ca^2+^ concentration leads to several inactivation mechanisms (step 3). GTP bound to the active Gα_olf_ subunit is hydrolyzed to GDP, inactivating it, and causing it to re-associate with the βγ subunits. Ca^2+^ binds to Calmodulin (CaM) which in turn activates CaMKII. Calmodulin potentially reduces CNG channel affinity for cAMP through interaction with the A-subunit of the channel (step 4). CaMKII inhibits the activity of AC3 by phosphorylating it, preventing the generation of cAMP. PDE1C is also activated by Ca^2+^ bound CaM and accelerates the hydrolysis of cAMP to AMP (step 5). GRK mediated phosphorylation of the OR (step 6). Intracellular Ca^2+^ concentration is re-established through the activity of ion exchangers (NCKX4 and possibly PMCAs), and the high intracellular Cl^−^ concentration is reestablished through NKCC1 activity and the closure of ANO2 channels (step 7).

There are several reviews describing vertebrate phototransduction (Fu and Yau, 2007; Yau and Hardie, 2009; Fain et al., 2010; Vinberg et al., 2018; Lamb, 2020), and olfactory transduction cascade in detail (Schild and Restrepo, 1998; Touhara and Vosshall, 2009; Kaupp, 2010; Pifferi et al., 2010; Antunes and Simoes De Souza, 2016; Boccaccio et al., 2021). Here, we will summarize the latest findings and highlight some of the gaps in knowledge. Important components of the prototypical transduction cascades are compiled in Table 1 and shown in Figures 2, 3.

**Table 1:** Overview and comparison table of the transduction cascades in vertebrates and invertebrates in vision and olfaction.

		**Invertebrate**	**Vertebrate**
Vision	Transduction compartment/volume (femtoliter, fL)	Microvillus/~*10^−2^*–*10^−3^*	Modified cilium (outer segment)/~*10–1,000*
	Receptor	r-opsin, G_q_-coupled GPCR	c-opsin, G_T_ (G_i/o_ family)-coupled GPCR
	Effector enzyme (E)	PLCβ4: synthesizes DAG, IP_3_; catabolizes PIP_2_	PDE6: hydrolysis of cGMP
	Second messenger (SM)	DAG/PUFA/PIP_2_/IP_3_	cGMP
	SM homeostasis	DAG kinase (DKG) and DAG lipase (DAGL) regulate breakdown of DAG.	Guanylyl Cyclase GC1 and GC2: synthesis of cGMP
	Transduction channel	TRP/TRPL	CNGA1/B1 (rod), CNGA3/B3 (cone)
	Sensitivity (unitary response)	~10 pA/~10 mV	~1 pA/1 mV
	Kinetics (time-to-peak, ms)	20 (*Drosophila*)-500 (nocturnal spider)	100 (cone)−500 (amphibian rod)
	Lifetime of GPCR* (t_GPCR*_), ms	–	40 (mouse)−400 (amphibian)
	Amount of E*s per GPCR*	~10 (*Drosophila*)	~50
	Lifetime of E* (t_E*_), ms	–	200 (mouse)−2,000 (amphibian)
	SMs hydrolyzed/synthesized	500 (*Drosophila*)	100–1,000
	Channels opened by single photon	~15	~250
Smell	Transduction compartment/volume (femtoliter, fL)	1–4 dendrites/cilia of OSNs are inside each hair-like sensillum (diameter ~1 μm; length ~10–20 μm)	Cilium, 0.4
	Receptor	Odorant receptor (OR), Ionotropic Receptor (IR), Gustatory Receptor (GR), *Drosophila* genome encodes ~60 OR receptors	G_olf_-coupled GPCR, hundreds or even >thousand different receptors
	Effector enzyme (E)	OR/IR can act as ligand-gated channel, potential regulation by G proteins.	Adenylate cyclase III (AC3)
	Second messenger (SM)	Directly ligand gated.	cAMP
	Transduction channel	OR-Orco, IR	CNGA2/A4/B1b
	Sensitivity (unitary response)	–	~0.05 pA/0.25 mV (assuming 5 GΩ input resistance), threshold for spike: ~40 odor molecules
	Kinetics (time-to-peak, ms)	~200 for ORs	~250 (mouse)
	Lifetime of GPCR* (t_GPCR*_), ms	N/A	1 or less potentially due to fast dissociation of the odor molecule from the receptor
	Amount of E*s per GPCR*	N/A	<1

*activated.

Both olfaction and phototransduction in vertebrates relies upon G protein-coupled receptors, involve divergent classes of transduction components. In phototransduction the absorption of a photon causes isomerization of chromophore (see Figure 1C), activating rhodopsin to Metarhodopsin II (R^*^). Active rhodopsin can activate several G proteins, transducins (G_T_), by catalyzing the exchange of GDP for GTP on its α subunit (Figure 2, step 1; Shichida and Morizumi, 2007). In olfactory transduction, an odorant binds to the G protein-coupled olfactory receptor (Figure 3, step 1; Buck and Axel, 1991; Mombaerts, 1999; Touhara, 2002), which then activates the G-protein, G_olf_ (Jones and Reed, 1989; Belluscio et al., 1998). The activated G_α_ subunits act upon different effector enzymes within the cells: in phototransduction PDEγ is displaced (Figure 2, step 2) thus decreasing levels of cGMP by increasing its' hydrolysis by active PDE^*^. This lowered level of cGMP in the PR outer segment closes CNG channels (Figure 2, step 3; Pugh and Lamb, 1990). Conversely in olfactory transduction, G_α*olf*_ activates adenylyl cyclase III (AC3, Figure 3, step 2; Pace et al., 1985) which catalyzes the generation of cAMP from ATP (Bakalyar and Reed, 1990; Wong et al., 2000), opening CNG channels (Figure 3, step 3; Nakamura and Gold, 1987; Brunet et al., 1996).

Interestingly, neurons in the VNO also utilize G protein-coupled receptors to detect pheromones. These can be divided into two sub-groups: Basal neurons express the Gα_o_, the receptors sharing sequence homology with metabotropic glutamate receptors (Matsunami and Buck, 1997; Ryba and Tirindelli, 1997). Whereas, the apical neurons express Gα_i2_, and the receptors share sequence homology with T2R bitter taste receptors (Dulac and Axel, 1995). Unlike both olfaction and phototransduction, VNO neurons rely on Trp2 channels in their signaling cascade (Leypold et al., 2002; Stowers et al., 2002).

One of the most notable differences between vertebrate PR and OSN transduction is that their activation leads either to a hyperpolarizing response in photoreceptors or a depolarizing response in olfactory neurons. This is due to the classes of G-protein and effector enzymes they express. Photoreceptor G_T_ protein, which is coupled to phosphodiesterase, belongs to the inhibitory G_i_ family whereas G_olf_ belongs to G_s_ subfamily of G-proteins that couple to adenylate cyclases (Roof et al., 1985; Gilman, 1987; Jones and Reed, 1989). While OSNs are relatively hyperpolarized in the absence of odors, with a range of reported V_m_ values from −90 to −30 mV (Schild and Restrepo, 1998), vertebrate photoreceptors are relatively depolarized in darkness (V_m_ ~-40 mV). Therefore, vertebrate photoreceptors need to maintain a large inward cation current in darkness, which dominates their energy consumption. As light closes CNG channels, increasing ambient illumination decreases the energy consumption of photoreceptors (Linsenmeier and Braun, 1992; Okawa et al., 2008; Ingram et al., 2020) whereas higher concentration of odors would be expected to increase energy expenditure in OSNs.

A hallmark of vertebrate phototransduction is its large gain achieved *via* multiple steps of amplification: (1) one R^*^ activates many G^*^s, (2) PDE^*^ hydrolyzes many cGMP molecules, and (3) co-operative binding of cGMP to the CNG channels (see numerical values in Table 1). This large amplification allows rod photoreceptors to detect single photons (Baylor et al., 1979). As vertebrate (rod) phototransduction became the model for GPCR signaling cascade, it was previously assumed that high amplification is a general feature of G-protein signaling. However, it is now known that an individual activated olfactory GPCR activates < <1 G_olf_ proteins, requiring tens of odor molecules to generate an action potential in a mouse OSN (Bhandawat et al., 2005; Ben-Chaim et al., 2011). This lower amplification is still true for vertebrate olfactory neurons despite the additional amplification mechanism unrelated to the GPCR signaling cascade. Namely, the influx of Ca^2+^ through open CNG channels also opens the calcium activated chloride ion channel ANO2 (Figure 3, step 3). This leads to chloride efflux and further depolarization of the cell due to the higher Cl^−^ concentration in OSNs compared to a typical mature neuron (Kaneko et al., 2004).

Interestingly, photoreceptors also express ANO2 channels that are expected to mediate a depolarizing current (i.e., Cl^−^ efflux) (Thoreson et al., 2003; Cia et al., 2005; Stöhr et al., 2009). Since light activation of photoreceptors leads to a hyperpolarizing response and closing of ANO2 channels, this chloride current would effectively promote light-induced V_m_ hyperpolarization, i.e., act as a positive feedback mechanism. However, Cl^−^ efflux has been shown to inhibit Ca^2+^ channels in photoreceptor terminals which is expected to provide a negative feedback in response to V_m_ depolarization (Thoreson et al., 2000). Indeed, a Ca^2+^-activated chloride current has been shown to mediate negative feedback in monkey cones to prevent a regenerative depolarization by Ca^2+^ influx in the terminal (Yagi and Macleish, 1994).

Whereas the rod and cone CNG channels are highly selective for cGMP (Fesenko et al., 1985; Yu et al., 1996), the olfactory CNG channel has similar affinity to both cGMP and cAMP (Nakamura and Gold, 1987; Zufall et al., 1994). These differences in the affinity to cyclic nucleotides are naturally important for efficient transduction of light and odorant signals that are mediated by cGMP and cAMP, respectively. However, since olfactory channels are sensitive to both cGMP and cAMP, they also have the potential to be regulated by cGMP. Indeed, a subset of OSNs use also a cGMP-mediated signaling pathway (Meyer et al., 2000), exemplifying that odorant transduction may be more diverse than phototransduction in vertebrates. This is not surprising considering the multitude of genes encoding ORs detecting a wide variety of odorants (Niimura and Nei, 2007) compared to typically only 2–5 different genes encoding visual pigment molecules tuned to detect different several wavelengths of light (Table 1).

The phototransduction pathway is generally well-conserved in vertebrates. However, ciliary PRs come in two forms, highly sensitive rods that can mediate dim light vision and less sensitive cones that rarely saturate and can mediate daytime color vision. It is thought that early primitive metazoans had cone-like ciliary photoreceptors (Fain et al., 2010; Lamb, 2013, 2020), making these the evolutionarily older cell type. Unlike most invertebrates that adopted rhabdomeric PRs with high sensitivity and a wide dynamic range (see below), vertebrates further developed highly sensitive ciliary rod photoreceptors by tuning the molecular properties and expression levels of the visual pigment and transduction components (Ingram et al., 2016; Lamb, 2020). Rod photoreceptors enable vision in dim light and are able to mediate energy-efficient vision (Fain et al., 2010) together with cones in the classical vertebrate duplex rod/cone retina, from single photons up to bright daylight (~10^7^ photons μm^−2^ s^−1^, Rodieck, 1998).

## Invertebrate Sensory Transduction Cascades

**Figure 4:**
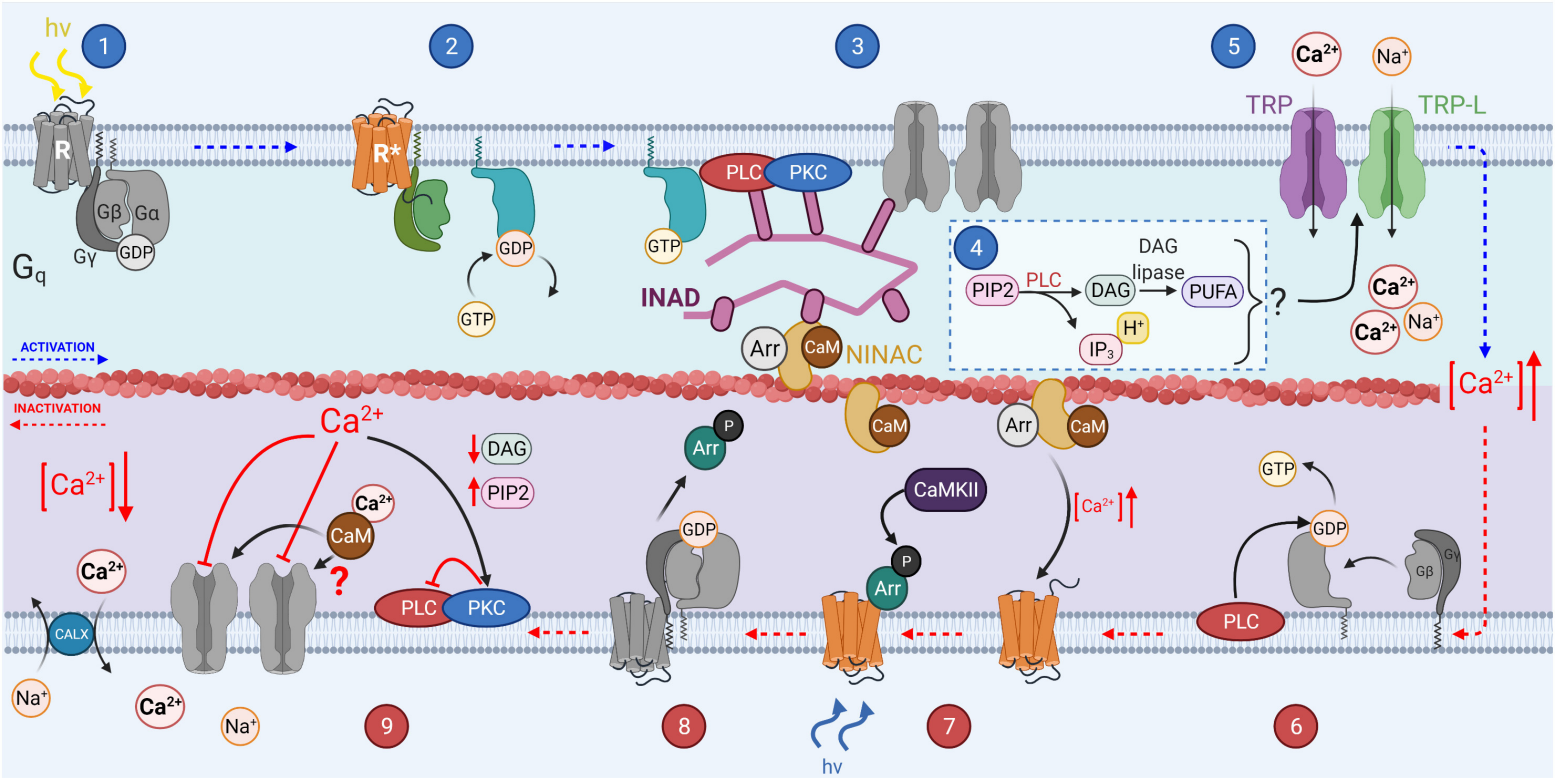
The invertebrate phototransduction cascade, inactivation, and adaptation mechanisms: A photon causes isomerization of the chromophore 3-hydroxy 11-*cis*-retinal to 3-hydroxy all-*trans*-retinal (step 1). This activates the opsin (step 2), which also activates the G_q_α subunit through GDP-GTP exchange. The G_q_α subunit then activates membrane bound PLC (step 3), which hydrolyzes PIP_2_ to produce IP_3_, DAG and H^+^ (step 4), and potentially DAG is further catalyzed to produce PUFAs. Some or all the products from step 4 activate the membrane bound TRP and TRP-L channels in step 5, causing an influx of Ca^2+^ and Na^+^ ions, depolarizing the microvillus. PLC can act as a GTPase, hydrolyzing the GTP bound to G_α_ to GDP (step 6). Invertebrate chromophore is bistable and is converted back from 3-hydroxy 11-*cis* retinal to 3-hydroxy-all-*trans* retinal at *via* the absorption of a second photon (step 7). CaMKII phosphorylates Arrestin 2 allowing it to dissociate from R* after the chromophore returns to its inactive state (step 8).

**Figure 5:**
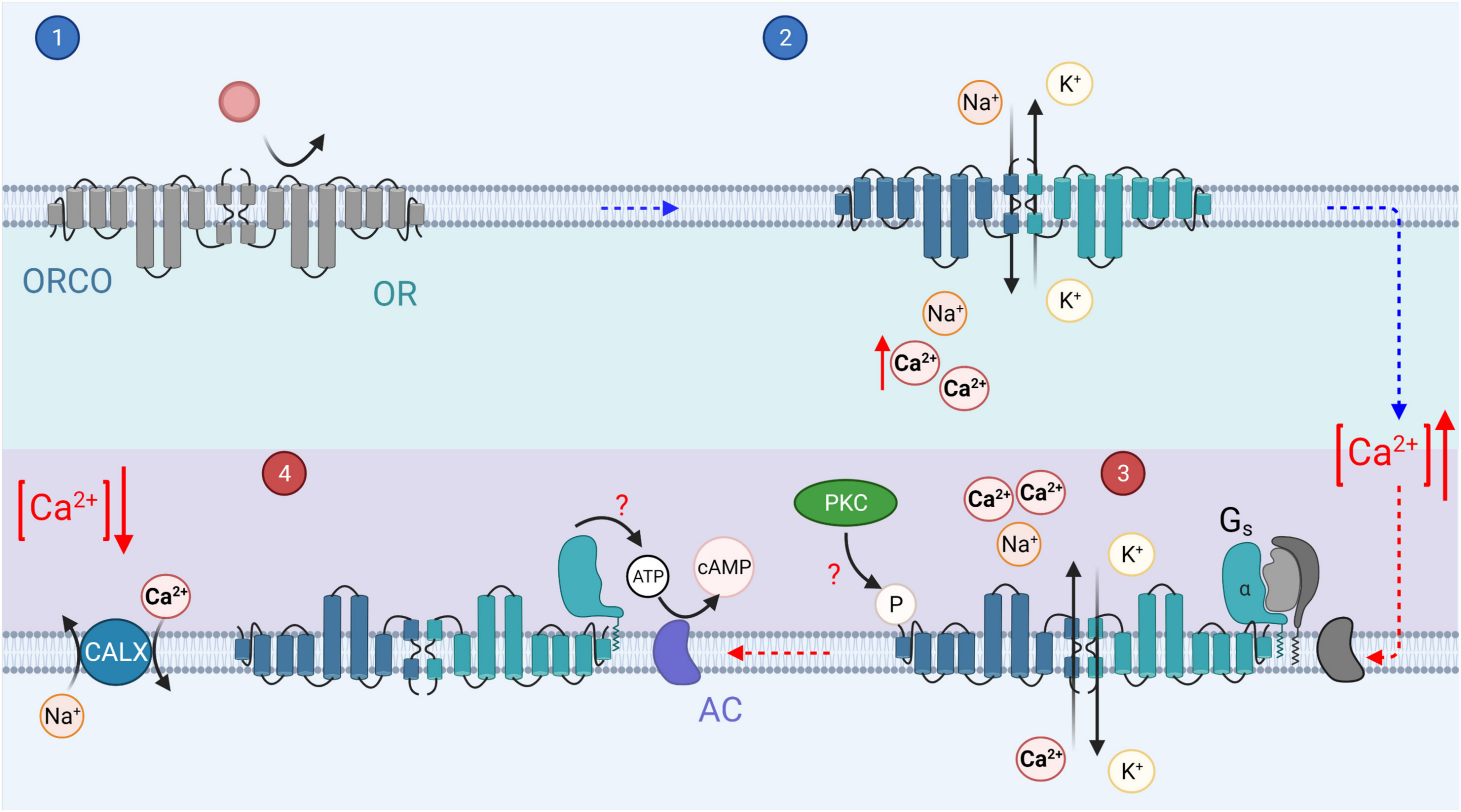
The invertebrate olfactory transduction cascade: In invertebrates, the ORs are 7 transmembrane domain receptors, which associate with a co-receptor, Orco. Upon binding of an odorant (step 1), the OR/Orco co-receptor channel pore opens, allowing the influx of cations, Ca^2+^ and Na^+^ (step 2). It is possible that a G protein mediated pathway regulates the sensitivity of the channels, odorant binding activates G_s_ (step 3). The G_s_α subunit then in turn activates adenylyl cyclase to create cAMP from ATP, which may mediate sensitization of the odorant response (step 4). Phosphorylation by PKC of the Orco receptor may mediate the olfactory neuron sensitization (step 3).

The invertebrate phototransduction cascade has also been reviewed extensively (Hardie, 2012; Montell, 2012; Hardie and Juusola, 2015), while olfactory transduction is still under active investigation (Touhara and Vosshall, 2009; Schmidt and Benton, 2020). In this review we will focus on *Drosophila* olfactory and visual transduction for simplicity, however several studies have identified many differences between invertebrate species including different receptors and transduction signaling mechanisms (for recent reviews, see Honkanen et al., 2017; Schmidt and Benton, 2020). The main steps of the transduction are shown in Figures 4, 5, and described below, with the main components listed in Table 1 for comparison. It should be noted that these Figures show pathways for which there is evidence in literature although the physiological relevance of some of them remain controversial. Furthermore, for the sake of simplicity they do not show all discovered pathways. Specifically, olfactory transduction in invertebrates appears to be extremely diverse as will be briefly discussed below.

Phototransduction is initiated when light induces a conformational change in the chromophore attached to an opsin (see Figure 1D for chromophore structure Figure 4, step 1; Paulsen and Schwemer, 1972). Unlike in vertebrates, the chromophore remains bound to opsin. Upon activation, rhodopsin is isomerized to metarhodopsin which activates the heterotrimeric G_q_ protein through GDP-GTP exchange, releasing the G_q_α subunit (Figure 4, step 2; Scott et al., 1995). G_q_α activates membrane-bound phospholipase C (PLC, Figure 4 step 3; Bloomquist et al., 1988; Estacion et al., 2001). PLC in turn hydrolyzes phosphatidylinositol 4,5-bisphophate (PIP_2_) to produce inositol 1,4,5-trisphosphate (IP_3_), diacylglycerol (DAG), and H^+^ in a classical second messenger pathway mediating a multitude of functions across multiple species (Figure 4, step 4; Litosch, 2015; Machaty, 2016; Martin-Aragon Baudel et al., 2020). Despite extensive efforts, the second messenger(s) gating Ca^2+^ permeable transient receptor potential (TRP) and transient receptor potential-like (TRP-L) cation channels *in vivo* remain unknown (Figure 4, step 5; Montell and Rubin, 1989; Hardie and Minke, 1992; Phillips et al., 1992; Huang et al., 2010). DAG itself is a well-known TRP channel activator and has been shown to activate recombinant TRPL channels (Estacion et al., 2001). However, DAG can also be converted into polyunsaturated fatty acids (PUFAs) by DAG lipase, and PUFAs are effective activators of both TRP and TRPL channels in intact *Drosophila* cells (see Figure 4, step 4; Chyb et al., 1999; Estacion et al., 2001). There is also significant evidence supporting a model in which activation of PLC leads to depletion of PIP_2_ in the phospholipid bilayer of microvilli, generating a mechanical force that leads to opening of TRP and TRPL (Hardie and Franze, 2012). Since DAG appears to be an ineffective activator of the native invertebrate TRP channels, it is most likely that either PIP_2_ depletion and/or PUFAs mediate the gating of invertebrate phototransduction channels in response to light. IP_3_ can also mediate Ca^2+^ release from ER but this mechanism does not contribute to *Drosophila* phototransduction (Acharya et al., 1997; Raghu et al., 2000). Invertebrate phototransduction has an amplification step not present in vertebrates which contributes to the very high sensitivity of rhabdomeric photoreceptors when compared even to rods (Hardie and Raghu, 2001; Fain et al., 2010). Once enough second messenger has accumulated during the variable latent period (15–100 ms) and the first TRP channel opens, the influx of Ca^2+^ mediates sensitization of the remaining TRP channels to their gating substance leading to the majority of the TRP channels in an individual microvillus opening even in response to a single photon (Henderson et al., 2000).

Invertebrate olfactory transduction appears to use a diverse set of pathways across species, within a single species, and even within a single OSN (for a recent review, see Schmidt and Benton, 2020). The receptors can be divided into two different classes; (1) seven transmembrane domain odorant receptors (ORs) (Clyne et al., 1999) and (2) three transmembrane domain ionotropic receptors (IRs, see Table 1; Benton et al., 2009). IRs have some homology with ionotropic glutamate receptors but will not be discussed extensively in this review. This diverse set of olfactory receptors may help animals to detect a large amount of different odorant molecules with high temporal resolution or high sensitivity across a wide range of background odorant levels. *Drosophila* odorant receptors (ORs) are odorant specific proteins (Clyne et al., 1999) with no obvious sequence homology to GPCRs (Benton et al., 2006) that form heteromers with a co-receptor, Orco [previously OR83b (Larsson et al., 2004; Butterwick et al., 2018)]. These OR/Orco complexes form ligand-gated cation channels (Figure 5, step 2; Sato et al., 2008; Smart et al., 2008; Wicher et al., 2008; Nakagawa and Vosshall, 2009). However, this type of ionotropic pathway is typically assumed to be relatively insensitive and not sufficient to mediate highly sensitive olfaction in flying insects. As a resolution to this apparent contradiction, odorant-binding was suggested to activate G_s_ and consequently induce production of cAMP molecules that presumably gate the Orco channel (Figure 5, step 4; Wicher et al., 2008; Miazzi et al., 2016). Indeed, as an example, vertebrate taste receptors can utilize either metabotropic GPCRs or ionotropic ligand-gated channels depending on the taste molecules (Kinnamon and Finger, 2019). However, experiments by Wicher et al. relied upon the HEK293 expression system, not directly demonstrating that G_s_-mediated signaling plays a role in intact insect OSNs. Indeed, a subsequent study by Andrea Yao and Carlson (2010) did not find a significant role for any GPCR signaling in *Drosophila* OR-mediated transduction. On the other hand, several other studies have lent support to the contribution of various metabotropic pathways to *Drosophila* olfactory transduction *in vivo* (Gomez-Diaz et al., 2004; Kain et al., 2008; Chatterjee et al., 2009; Deng et al., 2011; Getahun et al., 2013). Thus, the evidence taken as a whole suggests that invertebrate OR/Orco receptors mediate fast ionotropic transduction, but slower and potentially more sensitive metabotropic G protein coupled signaling cascade reminiscent of vertebrate olfactory transduction described above probably mediates the regulation of transduction (further described below) (Nakagawa and Vosshall, 2009; Hansson et al., 2010; Miazzi et al., 2016). Thus, the role of G protein mediated signaling in the invertebrate olfactory transduction is most likely to be a minor secondary one, perhaps solely regulatory.

Interestingly, an alternative way to increase the sensitivity of the invertebrate OSNs was suggested recently: a sodium channel (Pickpocket 25, PPK25) was shown to mediate Ca^2+^/calmodulin-dependent amplification of odorant responses in certain olfactory receptor neurons in *Drosophila* (Ng et al., 2019). This mechanism is analogous to vertebrate OSNs in which odorant induced Ca^2+^ influx activates ANO2 channels to promote depolarization (see above). In addition, a recent study showed that the OR/Orco complex can also be activated by odor-induced Ca^2+^ influx *via* a calmodulin mediated pathway in *Drosophila* olfactory neurons (Jain et al., 2021). This is analogous to invertebrate phototransduction where the signal is amplified *via* Ca^2+^ feedback (see above) and maybe critical for increasing the sensitivity of the OR-mediated olfactory transduction. In summary, it seems plausible that a combination of ionotropic and metabotropic signaling together with Ca^2+^ feedbacks and PPK25 channel can promote both fast and sensitive olfaction that is critical specifically for flying insects (Getahun et al., 2012, 2013).

## Inactivation of Signal Transduction

### Vertebrate

Timely inactivation of transduction is key to maintaining the temporal relevance of information from primary sensory neurons. Each activated component of the signaling pathway needs to be inactivated and the concentration of secondary messengers restored to pre-stimulus levels to enable rapid and repeated detection of sensory stimuli. In this section we will focus on the inactivation of receptors and G_α_/effector complex in vertebrate PR and OSN cilia since these processes are required for the cessation of the electrical PR and OSN response. Mechanisms that accelerate response recovery and/or regulate the sensitivity of the receptor cells to odors/light by modulating the homeostasis of second messengers and the affinity of cyclic nucleotides to CNG channels will be discussed below in the context of adaptation.

In *vertebrate phototransduction*, the active receptor, R^*^, is inactivated through two processes: rapid initial phosphorylation through the activity of *GPCR kinase* 1 (GRK1) (Bownds et al., 1972; Cideciyan et al., 1998; Lyubarsky et al., 2000; Sakurai et al., 2015) reducing R^*^'s catalytic activity (Figure 2, step 4; Xu et al., 1997), and rapid arrestin binding (Figure 2, step 5; Kuhn, 1978; Wilden, 1995; Kennedy et al., 2001). Rods, in mice, use only Arr1 whereas cones use both Arr1 and Arr4, however the functional significance of having two different isoforms in cones is not clear since deletion of either one of these arrestins alone does not have a significant physiological phenotype in mice (Lyubarsky et al., 2002; Shi et al., 2007; Nikonov et al., 2008). There is evidence that vertebrate olfactory receptors are also desensitized by GPCR kinase (GRK3, Figure 3, step 6) and that this is important for fast recovery of the olfactory response (Schleicher et al., 1993; Boekhoff et al., 1994; Peppel et al., 1997). However, the significance of GRK3 in modulating OSN desensitization has been called into question recently (Kato et al., 2014). In addition to GPCR kinase, second *messenger activated kinase* (PKA) is thought to play a role in rapid olfactory transduction termination potentially by phosphorylating the olfactory receptor (Figure 3, step 6; Boekhoff et al., 1992). However, if olfactory receptor phosphorylation plays a physiological role in terminating the olfactory response in vertebrates remains unclear since although Boekhoff et al. presented data supporting the role of PKA in fast response termination, their experiments did not confirm that PKA targets the receptor.

The active effector/G_T_α complex in the photoreceptors is inactivated through hydrolysis of bound GTP by GTPase-accelerating protein (GAP) complex (Figure 2, steps 6 and 7; Chen et al., 2000). Inactivation of the G_Tα_/PDE^*^ complex is the slowest step in mouse rod phototransduction termination and therefore rate-limits light response recovery after stimulation (Krispel et al., 2006). However, in amphibian cone photoreceptors inactivation of the receptor rather than PDE^*^ appears to rate-limit the light response recovery (Matthews and Sampath, 2010; Zang and Matthews, 2012). The rate-limiting step of mammalian cone phototransduction termination remains unknown. The active effector enzyme of olfactory transduction, AC3, is phosphorylated by calmodulin kinase II (CaMKII) to inhibit cAMP production (Wei et al., 1996). This inhibition is thought to contribute to the termination of olfactory transduction after odorant stimulation (Wayman et al., 1995; Wei et al., 1996, 1998). The mechanism that rate-limits the recovery of the electrical response of vertebrate OSN cilia appears to be mediated by the inactivation of the Ca^2+^-activated Cl^−^ channels, rather than any step of the GPCR signaling cascade, following the kinetics of Ca^2+^ extrusion from the OSN cilia (Reisert and Matthews, 2001; Antolin et al., 2010). Regulation of Ca^2+^ and potential modulation of the receptor or effector inactivation in PRs and OSNs by Ca^2+^ feedback will be discussed below.

### Invertebrate

Like in vertebrate phototransduction, inactivation of the active receptor (R^*^) in invertebrates involves Arrestins, two of which have been identified in *Drosophila*, Arr1 and Arr2 (Smith et al., 1990). However, unlike vertebrates, phosphorylation of rhodopsin is not necessary for termination of phototransduction or the binding of Arr2 in invertebrates (Plangger et al., 1994; Vinos et al., 1997). The G_q_α subunit itself may facilitate the binding of Arr2 to R^*^ (Hu et al., 2012). Binding of Arr2 displaces the G_q_α subunit from R^*^, reducing its activity (Figure 4, step 7). Interestingly CaMKII, a kinase that inhibits AC3 in vertebrate OSN, phosphorylates Arr2 in *Drosophila* (Matsumoto et al., 1994). However, this phosphorylation is not necessary for binding of arrestin to rhodopsin, but rather enables arrestin to dissociate from rhodopsin once it returns back to the ground state *via* absorption of a long-wavelength photon (Alloway and Dolph, 1999).

As in vertebrate phototransduction, the effector enzyme PLC functions as a GTPase activating protein (GAP) for G_qα_, mediating the termination of its activity (Figure 4, step 6; Cook et al., 2000). Inactivation of invertebrate phototransduction is significantly accelerated by Ca^2+^ feedback mechanisms which will be discussed below. As discussed earlier, invertebrate OSNs rely largely on GPCR-independent transduction where the receptor acts as an odor-gated channel. This allows fast inactivation of the odor-induced signal without the requirement for complicated enzymatic reactions to inactivate the multiple components involved. This strategy provides fast odor detection while conserving energy and can therefore be particularly advantageous for an insect with multiple predators and potentially scarce food sources. Though, specifically flying insects have also very sensitive olfaction that could be mediated by regulation of the OR/Orco complex by GPCR signaling cascade(s) as discussed above. However, the molecular details of these metabotropic pathways remain unclear and controversial.

## Adaptation Mechanisms in Invertebrate and Vertebrate Olfactory and Phototransduction Cascades

Animals live in environments where sensory exposure levels can vary dramatically, for example from the low light levels of shaded forests and underground burrows to the bright sunlight of a field at noon. The ability to adapt to these very dramatic differences in light levels is crucial for survival. Adaptation is a universal feature of all sensory systems, including vision and olfaction (Torre et al., 1995; Zufall and Leinders-Zufall, 2000); it is important to permit detection of novel stimuli in the presence of persistent activation. For vision, two crucial types of adaptation occur, light and dark adaptation. Here we define light adaptation as a deterministic and reversible process where the gain of the transduction can be adjusted up or down in response to increase or decrease in ambient light, respectively, to enable detection of increments or decrements of stimuli around the background stimulus level. Dark adaptation refers here specifically to visual adaptation to a large drop in ambient light following a bright light exposure that has bleached a significant fraction of the visual pigment molecules (Lamb and Pugh, 2004). This process involves regeneration of the visual pigment molecules, a process that can be quite slow specifically in vertebrate rod photoreceptors. This slow regeneration is responsible for the temporary inability to see you may have experienced when stepping into a dark movie theater. Both adaptation processes are critical for our ability to see over a wide range of light conditions, as well as adapt quickly to changes in ambient light during our everyday lives. Although both vertebrates and invertebrates can see over an impressive range of illumination conditions, they have adopted very different strategies to do so. Additionally, whereas increasing background light intensity decreases the sensitivity of photoreceptors based on Weber law, invertebrate olfactory receptors can be also sensitized by repetitive sub-threshold stimulation (Getahun et al., 2013; Mukunda et al., 2016). These differences will be highlighted below in terms of molecular mechanisms that control the gain and kinetics of the vertebrate and invertebrate phototransduction. Table 2 summarizes some of the important Ca^2+^ feedback mechanisms and adaptation characteristics across all four transduction modalities discussed in this review.

**Table 2:** Overview and comparison of the adaptation and feedback mechanisms in invertebrate and vertebrate phototransduction and olfactory transduction cascades.

		**Invertebrate**	**Vertebrate**
Vision	SM homeostasis	DAG kinase (DKG) and DAG lipase (DAGL) regulate breakdown of DAG.	Guanylyl Cyclase GC1 and GC2: synthesis of cGMP
	Calcium feedback	In microvilli: light ↑Ca^2+^ from 0.1 to 1,000 μM. In high Ca^2+^: first, positive feedback on TRP channel and IP_3_ receptor activation; Then, acceleration of inactivation of TRP channels potentially *via* PKC (shortens bump duration); CaM-mediated acceleration of GPCR* inactivation (*via* arrestin) and Ca^2+^-dependent PKC-mediated inactivation of PLC*.	Light ↓Ca^2+^ in the outer segment from ~0.5 to ~0.05 μM (rods) or ~0.005 μM (cones). In low Ca^2+^: GCAP1/GCAP2 accelerate cGMP synthesis, Ca^2+^-dependent acceleration of GPCR* and potentially PDE6*via recoverin.
	Calcium regulation	Diffusion, ER sequestration, Na^+^/Ca^2+^ exchange	Na^+^/Ca^2+^, K^+^ exchangers: NCKX1 (rod), NCKX2/4 (cone)
	Channel co-operativity	Ca^2+^-dependent sensitization of channels -> Thresholding: opening of 1 channel leads to abrupt opening of ~15 channels.	3
	Pigment regeneration/dark adaptation/vision in bright light	Bistable pigment that can be regenerated by long wavelength light but also in total darkness, regulation of refractory period by accelerating inactivation of GPCR*, PLC* and clearance of DAG and/or replenishment of PIP_2_ enable vision in bright light.	Visual cycles *via* RPE and/or Müller cells to regenerate visual pigment rate-limiting for dark adaptation and potentially required to prevent pigment depletion in bright light for cones.
	Light adaptation	Dynamic range: from single photon up to 10^5^-10^6^ photons/s. Ca^2+^ feedback by inactivation of GPCR* by arrestin, acceleration of GTPase activity of ${\text{G}}_{\text{q}}^{*}$ by PLC.	Dynamic range: Rods: 1–~1,000 photons/s; Cones: ~100–10^7^ photons/s. Ca^2+^ feedbacks; GRK-mediated phosphorylation and arrestin binding to GPCR*; acceleration of GTPase activity of G* by PDE6 and GAP complex (RGS9, Gb5).
Smell	Channel co-operativity		1.5
	Calcium feedback	Ca^2+^/CaM mediate positive feedback on OR-Orco and PPK25 Na^+^ channels.	Odor stimulation increases Ca^2+^ through CNG channels by several-fold from basal level of ~0.05 μM (in the absence of odorants). In high Ca^2+^: Positive feedback to activate Ca^2+^-activated Cl^−^ channels; Negative feedback: CaMKII-mediated inhibition of ACIII; Ca^2+^/CaM-mediated activation of PDE1C.
	Calcium regulation	CALX	Na^+^/Ca^2+^, K^+^ exchanger: NCKX4
	Adaptation	Dephosphorylation of Orco.	Ca^2+^ feedback; Phosphorylation by cAMP-dependent kinase PKA.

*activated; ↓decreased.

## Light/Odor Adaptation

### Regulation of Ca^2+^ in the Transduction Compartments

Calcium ions (Ca^2+^) play a critical role in both light and odor adaptation. Thus, it is important to first understand how Ca^2+^ is regulated in the cilia or microvilli of the photoreceptors and OSNs in response to sensory stimulus. The channels mediating the influx of Ca^2+^ into vertebrate PR and OSN cilia are CNG channels which are not only permeable to Na^+^ but also to Ca^2+^ (Yu et al., 1996). In invertebrates, the transduction channels, TRP and OR/Orco receptor channels, also mediate Ca^2+^ influx into the PR and OSN transduction compartments (Chu et al., 2013). A sensory stimulus leads to increases in cytosolic Ca^2+^ in all OSNs and in invertebrate PRs (i.e., activation = increase in neuronal Ca^2+^). However, vertebrate photoreceptors are a notable exception to this rule, their CNG channels are closed by light, leading to a decline in the outer segment [Ca^2+^]. Even if the details of how Ca^2+^ is regulated in these different transduction compartments may differ, the general principle of using the transduction channel to regulate Ca^2+^ influx seems to be a conserved strategy. This way Ca^2+^ level in the cilia or microvilli can be proportional to sensory stimulus level, an important feature for its ability to adjust the transduction gain in response to changes in ambient light or odorant level.

In vertebrate PR outer segments [Ca^2+^] is ~0.5 μM in darkness and in bright light decreases 10-fold in rods and 100-fold in cones (see Table 2, Sampath et al., 1998, 1999; Woodruff et al., 2002). In the small microvilli of the invertebrate PR [Ca^2+^] can quickly increase 10,000-fold upon light stimulation (Asteriti et al., 2017), a several log-units larger dynamic range compared to vertebrate rods and cones (See Table 2 for values). Additionally, the global Ca^2+^ levels in PR cells can range from ~0.1 μM in darkness up to 10 μM in bright light (Oberwinkler and Stavenga, 2000). This could play a role in the larger adaptation capacity of individual invertebrate microvillar photoreceptors compared to ciliary photoreceptors found mainly in vertebrates. The basal Ca^2+^ level in vertebrate olfactory neurons is ~0.05 μM (Leinders-Zufall et al., 1997), and can increase several-fold in response to odor stimulation (Antolin et al., 2010). However, this increase is not as large as in photoreceptors, which are known for their remarkable ability to adapt. Permeability of the transduction channels to Ca^2+^ and particularly the fraction of inward current carried by Ca^2+^ may play an important role for the amplitude of Ca^2+^ signals. For example, Ca^2+^ ions carry a significantly smaller fraction of the transduction channel current in rods than in cones (Picones and Korenbrot, 1995), microvillar photoreceptors (Chu et al., 2013) or vertebrate OSN CNG channels (Frings et al., 1995). This can contribute to the higher fluxes of Ca^2+^ across the plasma membrane to promote faster and larger Ca^2+^ signals and adaptation in vertebrate cones or fly photoreceptors compared to rods (Sampath et al., 1998, 1999).

In addition to transduction channels, an important part of Ca^2+^ homeostasis is its cytosolic clearance. Visual and olfactory transduction in both vertebrates and invertebrates all use plasma membrane Na^+^/Ca^2+^ exchangers to extrude Ca^2+^. Na^+^/Ca^2+^ exchangers have significantly higher capacity compared to PMCAs. This enables faster regulation of Ca^2+^, however, the affinity of Na^+^/Ca^2+^ exchangers to Ca^2+^ is rather low and they are not able to drive Ca^2+^ to extremely low levels. In some cases, plasma membrane ATPases (PMCAs) (Weeraratne et al., 2006; Castillo et al., 2007) as well as intracellular stores (ER and mitochondria) (Molnar et al., 2012; Bisbach et al., 2020; Hutto et al., 2020; Liu et al., 2020) can also contribute to cytosolic Ca^2+^ homeostasis in sensory cilia or microvilli, but the contribution of these mechanisms to the overall Ca^2+^ homeostasis and transduction is not well-understood.

In vertebrate photoreceptors extrusion of Ca^2+^ is directly related to the kinetics of response termination and light adaptation, as it mediates the reduction of Ca^2+^ required for critical feedback mechanisms that accelerate response termination and desensitize the photoreceptors. Extrusion of Ca^2+^ from the vertebrate OSN cilium directly controls inactivation of the Ca^2+^-activated Cl^−^ channels and thus rate-limits the recovery of the electrical response of olfactory neurons (Antolin et al., 2010). On the other hand, in both invertebrate phototransduction and olfactory transduction, Ca^2+^ extrusion plays a role in Ca^2+^ homeostasis rather than directly contributing to adaptation kinetics in response to increases in ambient light or odorant levels.

Vertebrate PR and OSN cilia use Na^+^/Ca^2+^, K^+^ exchangers (NCKXs, for a recent review Jalloul et al., 2020) to extrude Ca^2+^. These exchangers use energy derived from the driving force of both Na^+^ and K^+^ to extrude Ca^2+^ and therefore, can promote fast Ca^2+^ extrusion, response recovery or adaptation. Their role in vertebrate rod and cone photoreceptors was recently reviewed in Vinberg et al. (2018). Rod photoreceptor outer segments rely almost exclusively on NCKX1 for Ca^2+^ extrusion (Reilander et al., 1992; Vinberg et al., 2018) although some other mechanism(s) may also have a small contribution to Ca^2+^ homeostasis in the mouse rod outer segment (Vinberg et al., 2015; Bisbach et al., 2020). Cones were originally thought to rely exclusively on NCKX2, however, a recent study showed that vertebrate cones (including primate cones) also express NCKX4 in their outer segments and that NCKX4 is important for the fast termination and light adaptation of mouse cones (Vinberg et al., 2017). It has been speculated that cones use different exchangers and/or two exchangers to fulfill their requirement for faster signaling and wider operating range. However, a recent study did not find any significant biophysical differences between different NCKX isoforms (Jalloul et al., 2016). A more relevant question might be why rods acquired NCKX1 instead of NCKX2 and NCKX4, which are used by the evolutionarily older cone photoreceptors. It is possible that it could be related to how these exchangers can interact with CNG channels. It is known that NCKX1 forms heteromers with the rod CNG channel (Bauer and Drechsler, 1992), and the same may be true also for the cone channels and exchangers (Kang et al., 2003). Interestingly, NCKX4 is also important for termination and adaptation of the mouse OSN response (Stephan et al., 2011) although OSNs also express functionally important PMCA2 and PMCA4 in their cilia (Weeraratne et al., 2006; Castillo et al., 2007).

The identity of the plasma membrane Na^+^/Ca^2+^ exchangers in the *Drosophil*a PR and OSN cilia and microvilli is also known. Interestingly, both of these sensory neurons appear to rely on Na^+^/Ca^2+^ exchanger CALX, a homolog of vertebrate exchanger NCX (Wang et al., 2005; Halty-Deleon et al., 2018). However, CALX has a unique property of being inhibited by high cytosolic Ca^2+^ (Hryshko et al., 1996), a property that is puzzling considering its presumed role in restoring low Ca^2+^ after stimulus-induced increase of [Ca^2+^]_i_. A recent study found that CALX is also expressed in the ER of *Drosophila* photoreceptors and contributes to increased ciliary Ca^2+^ after light stimulation by transporting Ca^2+^ from ER stores (Liu et al., 2020). Although it might be expected that invertebrate microvillar photoreceptors need extremely efficient mechanisms to extrude Ca^2+^ due to their fast response kinetics and wide operating range, it should be pointed out that invertebrate phototransduction is also compartmentalized to extremely small microvilli, where the influx/efflux of just one calcium ion can raise or decrease [Ca^2+^]_i_ by 1 μM. Thus, the large membrane surface area to volume ratio may be more important for fast and efficient Ca^2+^ signaling than the biophysical properties of the exchanger protein itself.

### Regulation of Receptor Inactivation

Both vertebrate and invertebrate phototransduction employs Ca^2+^ feedback to accelerate inactivation of the active receptor, R^*^. However, the mechanisms and their relative contributions to the lifetime of R^*^ and light response termination are different. The lifetime of R^*^ is ~40 ms in mammalian (Gross and Burns, 2010) and ~400 ms in amphibian dark-adapted photoreceptors (Nikonov et al., 1998). The mechanisms that contribute to these relatively short lifetimes: phosphorylation by GRK1 and binding of arrestin have already been discussed in this review. R^*^ lifetime is further shortened by background light *via* Ca^2+^ feedback mediated by recoverin in both rods and cones (Xu et al., 1997; Cideciyan et al., 1998; Lyubarsky et al., 2000; Sakurai et al., 2015). In the dark state, where intracellular calcium is high, Ca^2+^ bound recoverin inhibits the activity of GRK1 (Kawamura, 1993; Chen et al., 1995; Klenchin et al., 1995). Once light closes CNG channels, and intracellular calcium is reduced, recoverin no longer binds calcium and thus releases inhibition of GRK allowing it to phosphorylate R^*^. Thus, increasing light intensity is expected to decrease the lifetime of R^*^, and the subsequent gain of the phototransduction. Since the lifetime of R^*^ is not rate-limiting for the response recovery in mouse rods, its role in shaping the light responses is not obvious, but it regulates kinetics of mouse rod bright flash responses (Makino et al., 2004) and light sensitivity of cones (Sakurai et al., 2015). Moreover, the contribution of recoverin to light adaptation at least in rod photoreceptors is minor, affecting only adaptation kinetics but not the steady state adaptation (Chen et al., 2010; Morshedian et al., 2018).

Although phosphorylation of the R^*^ in invertebrate PRs is less important, arrestins also have a critical role in inactivating the R^*^ in microvillar photoreceptors as described above. Whereas, Arr1 and Arr4 in vertebrate photoreceptors are not known to be regulated by any Ca^2+^-dependent mechanism, Arr2 is regulated by Ca^2+^/calmodulin in *Drosophila* (Kahn and Matsumoto, 1997). In low calcium solutions inactivation of R^*^ is slowed down by 10-fold, and Ca^2+^ dependent inactivation is abolished when calmodulin (CaM) or CaM-binding myosin III (NINAC) is mutated (Li et al., 1998; Liu et al., 2008), indicating that Ca^2+^ influx accelerates the binding of Arr2 to R^*^via CaM, and NINAC sequesters Arr2 in low Ca^2+^. This suggests that whereas Ca^2+^ feedback on R^*^ inactivation has minimal significance in vertebrate photoreceptors, it has a major role in accelerating light responses in *Drosophila*. This difference could be contributing to the faster light responses and vision in insects as compared to vertebrates.

The lifetime of active olfactory receptor in vertebrates is very transient, potentially as short as 0.1 ms. This has important implications for the gain and sensitivity of olfactory transduction which, unlike phototransduction, is not capable of transducing single odor molecule events (Ben-Chaim et al., 2011). Although there is some evidence that phosphorylation of the OR in vertebrates by GRK3 may be important for the fast inactivation of the activated receptor (see above), it is not known if any Ca^2+^ feedback mechanisms exist to regulate the lifetime of the active ORs. It may not be necessary since the baseline lifetime is already extremely short and determined by the short lifetime of the odorant-OR complex rather than by any enzymatic reaction (Bhandawat et al., 2005). As described above, sensitivity of invertebrate OR/Orco receptor is modulated by phosphorylation or dephosphorylation. Furthermore, as described above the sensitivity of the OR/Orco complex can be increased by Ca^2+^-calmodulin to mediate sensitization of the OSNs in response to weak stimulation (Mukunda et al., 2016; Jain et al., 2021).

### Regulation of Effector Enzyme Inactivation

Inactivation of the G_Tα_/PDE^*^ in vertebrate PRs is significantly accelerated by a GAP complex (see above). Initially, the lifetime of PDE^*^ was thought to be independent of background light level or Ca^2+^ (Nikonov et al., 1998). However, it has been shown in salamander and mouse rods that the lifetime of PDE^*^ shortens under increasing background light intensity, and experiments in mice suggest that this is mediated by recoverin (Nikonov et al., 1998, 2000; Chen et al., 2015; Morshedian et al., 2018). These results suggests that recoverin can mediate a Ca^2+^-dependent acceleration of R^*^ and PDE^*^ inactivation, though the contribution of this regulation to light adaptation capacity of vertebrate rod photoreceptors is very small.

In invertebrate PRs the inactivation of effector enzyme PLC is also mediated by Ca^2+^, but *via* different mechanisms than in vertebrates (Smith et al., 1991). In *Drosophila* PRs, light-induced increase of Ca^2+^ inhibits PLC through the kinase activity of PKC, slowing down depletion of PIP_2_ and synthesis of DAG (Figure 4, step 9; Shieh et al., 1997; Gu et al., 2005). This mechanism is expected to contribute to acceleration of the light response termination and desensitization of the microvillar photoreceptors upon increased ambient light, i.e., adaptation.

The effector enzyme in *vertebrate OSNs* is cAMP-synthesizing enzyme AC3, which is activated by G_α*olf*_ as described earlier. Restoration of resting state levels of cAMP after odor-induced increases is accelerated by inhibition of AC3 *via* a Ca^2+^/calmodulin kinase II mediated phosphorylation (Wei et al., 1996, 1998). Thus, inhibition of AC3 causes the accelerated termination of the electrical odor response, and the OSN is desensitized in response to increased Ca^2+^ concentration triggered by increased background odorant levels.

### Regulation of the Second Messenger Homeostasis

In addition to inactivating the effector enzyme of the GPCR signal cascade, the restoration of pre-stimulus second messenger concentration requires enzymes that either reduce the excess cAMP in vertebrate OSNs (Boekhoff and Breer, 1992) or DAG/PUFA in invertebrate PRs, respectively, or synthesize cGMP in vertebrate PRs or PIP_2_ in invertebrate PRs. Whereas, these enzymes are not directly part of the GPCR signaling cascade, some of them are known to play an important role in transduction termination and light adaptation. Indeed, regulation of cGMP synthesis by guanylyl cyclases (GC1 and GC2 in mice) is the dominant mechanism for regulating sensitivity in vertebrate rods and cones (Mendez et al., 2001; Sakurai et al., 2011). In darkness when Ca^2+^ is high, Guanylyl Cyclase Activating Proteins (GCAP1 and GCAP2 in mice) have Ca^2+^ bound to them and they cannot activate GCs. With light-induced decreases of Ca^2+^, Mg^2+^ replaces Ca^2+^ in GCAP proteins, and the Mg^2+^-GCAPs increase cGMP synthesis by activating GCs (Peshenko and Dizhoor, 2007). GCAP1 and GCAP2 have different affinity to Ca^2+^ so that GCAP1 is disinhibited and can activate GCs with smaller decline of Ca^2+^ in dim light whereas GCAP2 becomes more important under bright light conditions with larger decrease in Ca^2+^ (Makino et al., 2012). Invertebrate phototransduction is more complicated and as the identity of second messenger is still unclear, the contribution of its degradation/synthesis regulation to the light adaptation capacity is also not known. As described above the effector enzyme PLC plays an important role in invertebrate phototransduction since it releases DAG from PIP_2_. Whether enzymes like DAG lipase that convert DAG into PUFAs, or those involved in PUFA metabolism or PIP_2_ synthesis are regulated by light-induced increases of Ca^2+^ remain open questions.

Olfactory neurons in vertebrates express cAMP-hydrolyzing enzyme PDE1C specifically in their cilia (Yan et al., 1995), and PDE4A throughout the OSN excluding the cilium (Juilfs et al., 1997) which balance the synthesis of cAMP by AC3. Although PDE4A is not expressed in the OSN cilia, termination of mouse OSN response is only slowed when both PDE1C and PDE4A are knocked out (Cygnar and Zhao, 2009). However, odorant adaptation in mice lacking PDE1C alone is compromised, demonstrating a role for PDE1C in regulating the gain of the olfactory transduction in response to increased background levels of odorants (Cygnar and Zhao, 2009). PDE1C is known to be activated by Ca^2+^-bound calmodulin (Yan et al., 1995). Thus, it is expected that odorant-induced increase of Ca^2+^ activates PDE1C *via* Ca^2+^/calmodulin to accelerate hydrolysis of cAMP, explaining how PDE1C can contribute to adaptation.

In invertebrate olfactory transduction, increasing evidence has demonstrated that although G protein mediated signaling does not have a primary role in olfactory transduction, it is likely to be involved in the adaptation mechanisms. Odorant-binding has been suggested to activate G_s_ and consequently induce production of cAMP molecules that presumably gate Orco channel, perhaps as a mechanism to mediate the sensitization of the neurons (Figure 5, steps 3 and 4; Wicher et al., 2008; Miazzi et al., 2016).

### Regulation of the Transduction Channel

All transduction channels discussed in this review, including the ionotropic OR/Orco receptor channels, have been suggested to be regulated in response to changes in background sensory stimulus level. The CNG channels used by vertebrate PRs and OSNs as well as TRP channels in the invertebrate photoreceptors all have a binding domain for calmodulin. Indeed, *in vitro* studies suggested that the affinity of vertebrate PR and OSN CNG channels to second messengers is regulated by calmodulin mediated Ca^2+^ feedback (Hsu and Molday, 1994; Liu et al., 1994; Bradley et al., 2001; Song et al., 2008; Nache et al., 2016). However, Ca^2+^/calmodulin modulation may not be critical for vertebrate photoreceptor (Haynes and Stotz, 1997; Chen et al., 2010) or OSN adaptation (Song et al., 2008). The odor receptor-channel in invertebrates can also be regulated in response to increased background levels of odorants. Interestingly, phosphorylation (Sargsyan et al., 2011; Getahun et al., 2013) and dephosphorylation (Guo et al., 2017) of the Orco co-receptor sensitizes and desensitizes the *Drosophila* olfactory transduction in response to OSN activation, respectively. Phosphorylation is mediated by PKC but the molecular mechanism of dephosphorylation is not known. These results indicate that phosphorylation of the ORs by PKC mediates the sensitization of the ORNs to sub-threshold stimulation whereas dephosphorylation in response to stronger background odorant levels would mediate desensitization of the ORNs. These mechanisms together can contribute to widen the operating range of the insect olfaction (Jain et al., 2021). It is possible that calmodulin independent Ca^2+^ feedbacks on the CNG channels exist in vertebrate photoreceptors. For example, in catfish cone photoreceptors calmodulin does not modulate cone CNG channel activity (Haynes and Stotz, 1997). On the other hand, striped bass cones express CNG modulin that may be responsible for the Ca^2+^ induced modulation of CNG channel activity in vertebrate cones (Rebrik et al., 2012). The mammalian homolog to CNG modulin is echinoderm microtubule-associated protein-like 1 (Eml1) which is expressed in the human and mouse retina (Rebrik et al., 2012). It remains to be determined whether it plays any role in the mammalian cone or rod phototransduction. As described above, termination of the vertebrate olfactory transduction requires also closure of Ca^2+^-activated Cl^−^ channels. Gating of the Ca^2+^-activated Cl^−^ channel appears to be modulated *via* a Ca^2+^-dependent pathway mediated by calmodulin (Yang et al., 2014). Both TRP and TRP-L channels in invertebrate photoreceptors have CaM binding sites (Phillips et al., 1992; Chevesich et al., 1997), and have been hypothesized to contribute to light-adaptation. In summary, although a lot of *in vitro* evidence supporting Ca^2+^-dependent modulation of the transduction channels in vertebrate and invertebrate sensory transductions exist, its physiological significance *in vivo* is either minor or remains to be determined.

## Dark Adaptation and Pigment Regeneration in Vertebrate and Invertebrate Photoreceptors

**Figure 6:**
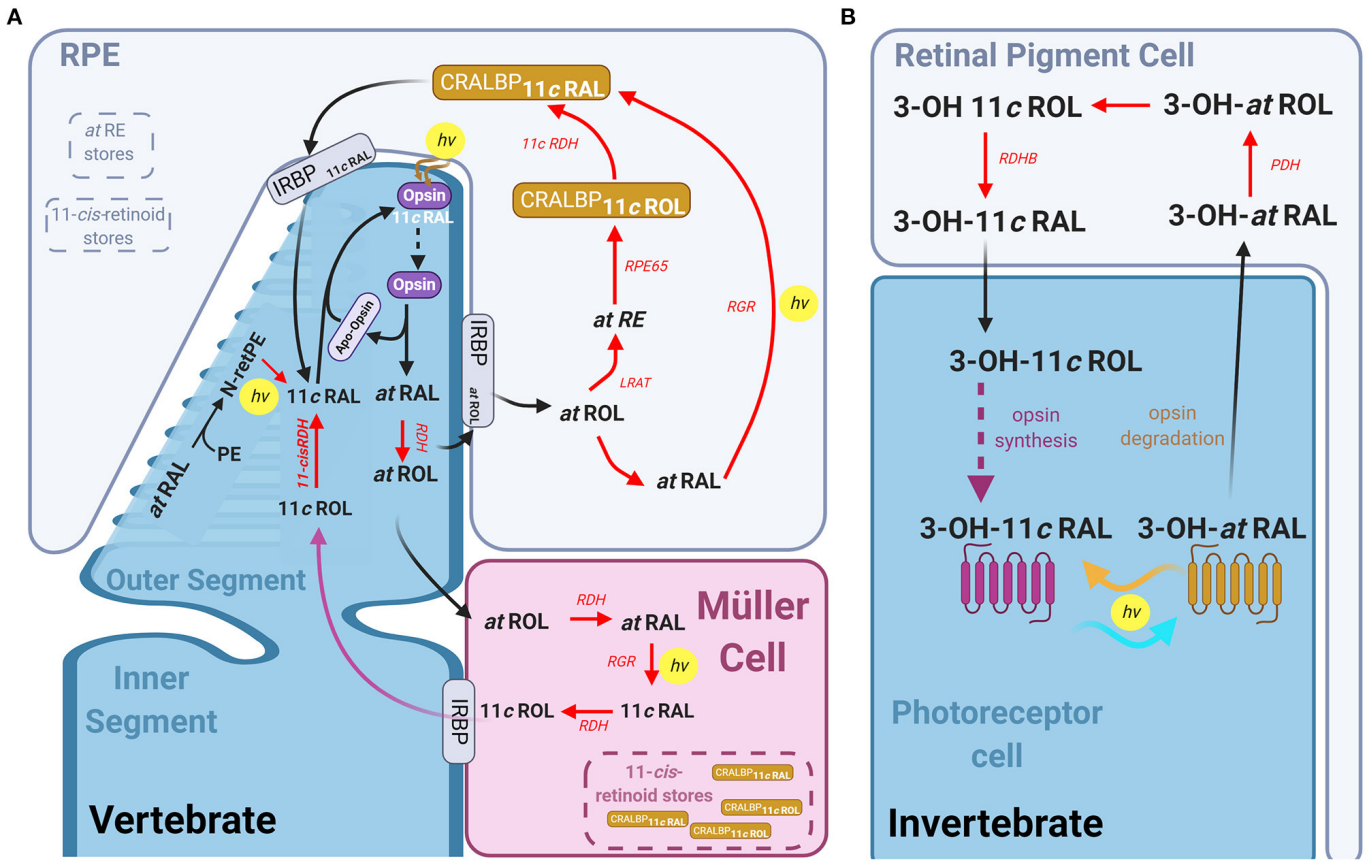
The visual pigment cycle reactions in vertebrate and invertebrate photoreceptors: **(A)** Pathways for visual pigment regeneration in the vertebrate retina, including both the classical and non-classical pathways. A cone photoreceptor (dark blue) and its' interactions with the muller glial cells (magenta) and Retinal pigment epithelium (RPE, light blue). Red arrows with red text annotation indicate enzyme mediated reactions and their respective enzymes. The classical pathway occurs *via* the RPE, all-*trans*-retinol is taken up by IRBP into the RPE, where LRAT, RPE65, and 11-*cis*-RDH mediates its conversion to 11-*cis*-retinal which is then taken up by the photoreceptor *via* IRBP again, which reassociated with the apo-opsin. The intrinsic light mediated pathway combining all-*trans*-RAL with phosphatidylethanolamine (PE) to form all-*trans*-N-retinyl-PE (N-retPE), which is then converted to 11-*cis-*retinal *via* a blue photon. Light mediated pigment regeneration can occur both in Müller cells and RPE, whereby all-*trans* retinal is converted to 11-*cis* retinal mediated by RGR opsin and the absorption of a photon. Stores of 11-*cis*-retinoids as well as all-*trans*-RE are present in both the RPE and Müller cells. **(B)** Summary of the visual pigment cycle in invertebrates. The invertebrate visual pigment, 3-OH 11-*cis* retinal is a bistable pigment, and remains associated to the opsin, upon absorption of a second photon, it can convert back from 3-OH all-*trans* retinal to 3-OH 11-*cis* retinal. An external pathway is only required in conditions in which the animal is calorie restricted. This pathway is mediated by the retinal pigment cells that surround the rhabdomeres and involves the Photoreceptor retinol dehydrogenase (PDH) and retinol dehydrogenase B (RDHB) enzymes.

Maintenance of vision during sustained background light or adaptation to darkness after bright light exposure requires a mechanism to reset the activated visual pigment back to its ground state. Invertebrate rhabdomeric photoreceptors can regenerate photopigment in a light-dependent manner. Their rhodopsin is bistable as the chromophore remains bound to opsin after the absorption of an initial photon, so that a further absorption of a long-wavelength photon converts the activated rhodopsin back to the ground state (see Figure 6B). This may imply that a separate pigment regeneration pathway as described below for vertebrates is not required for invertebrate vision in bright light. However, in case of limited nutritional intake, or when rhodopsin is endocytosed, a pigment regeneration pathway exists to allow the maintenance of normal chromophore (Wang et al., 2010). Indeed, a retinal dehydrogenase present in the retinal pigment cells that surround the photoreceptors, was found to promote synthesis of 3-OH-11-*cis*-retinal (Wang et al., 2012). This pathway could also be important for dark adaptation or under short-wavelength illumination when the photons for converting *cis*-retinal back to *trans*-retinal are not available.

Unlike the r-opsins in rhabdomeric photoreceptors, the c-opsins in ciliary photoreceptors are not thought to be bistable. Thus, pathway(s) to regenerate visual pigment are critical for vertebrate photoreceptors to escape saturation under continuous illumination or to restore full sensitivity after bright light exposure. Absorption of a photon by 11-*cis*-retinal (11cRAL) causes several conformational changes in rhodopsin converting it to a physiologically active meta II form (or R^*^) which eventually decays to free opsin and all-*trans* retinal (Kolesnikov et al., 2003; Lamb and Pugh, 2004). This process takes minutes in rods, but only seconds in cones (Shi et al., 2007; Estevez et al., 2009). Although formation of free opsin is a prerequisite for the pigment regeneration, restoration of light sensitivity after bright light exposure is thought to be rate-limited by the 11-*cis*-retinoid supply to the photoreceptors (Lamb and Pugh, 2004). Several pathways to deliver 11-*cis*-retinoids to rods or cones are now known to take place in retinal pigment epithelium (RPE), Müller and photoreceptor cells. These pathways have been reviewed previously (Lamb and Pugh, 2004; Wang and Kefalov, 2011; Kiser et al., 2014; Palczewski and Kiser, 2020) and are summarized in the following paragraphs and in Figure 6A.

Recycling of atRAL according to the canonical pathway occurs outside the photoreceptor, in the retinal pigment epithelium (RPE) monolayer (Dowling, 1960). Following activation, atRAL is reduced to all-*trans*-retinol (atROL) by retinol dehydrogenase (RDH) and transported to RPE by interphotoreceptor retinoid binding protein (IRBP). Lecithin:retinol acyltransferase (LRAT) esterifies the atROL to all-trans-retinyl-ester (atRE) (Macdonald and Ong, 1988; Saari and Bredberg, 1989). These esters are then hydrolyzed and isomerized to 11-*cis*-ROL (11cROL) by retinoid Isomerohydrolase (RPE65, Gollapalli and Rando, 2003; Moiseyev et al., 2003; Mata et al., 2004), and oxidized to 11cRAL by 11-*cis*-RDH. This 11cRAL is then transported into the photoreceptors by IRBP and re-associates with the apo-opsin to complete the cycle.

### Cone-Specific Pigment Regeneration Pathway Mediate by CRALBP

Critically, the canonical pigment regeneration pathway is too slow to resupply cones with sufficient pigment to remain responsive under continuous daylight conditions. In fact, several studies in multiple vertebrates found that cone sensitivity and pigment regeneration is maintained in the absence of RPE (Goldstein, 1970; Bruch Goldstein and Wolf, 1973). Thus, evidence overwhelmingly supports the existence of a cone-specific intra-retinal pigment regeneration pathway that supplies chromophore exclusively to cones. Studies in cultures of Müller cells from chicken found that they were able to synthesize 11cROL and 11-*cis* retinyl palmitate from atROL (Das et al., 1992), and CRALBP stimulates this synthesis (Mata et al., 2002). This is significant as it suggests a second population of cells in the retina that has isomerase activity (like RPE65 in the RPE) and retinyl ester synthase activity (Das et al., 1992). Pharmacological ablation of Müller cells in salamander, mouse, macaque or human retinas eliminates the ability of their cone photoreceptors to dark adapt following a bright light exposure in the absence of RPE (Wang and Kefalov, 2009; Wang et al., 2009). A later study demonstrated that CRALBP in Müller cells is critical for complete cone dark adaptation in isolated mouse retinas (Xue et al., 2015). It was initially proposed that CRALBP required interaction with Des1 in Müller cells to regenerate pigment in cones (Kaylor et al., 2014), however, deletion of *Des1* in the mouse Müller cells had no effect on cone dark adaptation in isolated retinas (Kiser et al., 2019). One hypothesis, proposed by Kiser et al. (2018), is that 11-*cis* retinoids bound to CRALBP in Müller cells form a 11-*cis* retinoid storage that can be quickly used for rapid pigment regeneration and dark adaptation of cones.

### Photic Pigment Regeneration Pathways

Recent work has potentially identified a light-dependent pathway for pigment regeneration in ciliary photoreceptors that depends on blue light. Much like the bistable pigment regeneration in rhabdomeres, this mechanism is independent of enzyme activity, and occurs in the outer segment membrane. After atRAL condenses with phosphatidylethanolamine (PE) to form all-trans-N-retinyl-PE in the lipid membrane, and a blue photon then converts this to 11-*cis*-N-retinyl-PE, which spontaneously hydrolyses to 11cRAL. Exposure of retinas to light around 450 nm wavelength after a bleach produced a higher regeneration of pigment than retinas left to regenerate in the dark (Kaylor et al., 2017). This mechanism also contributed to mouse photoreceptor pigment regeneration *in vivo* and is expected to be functional in both rods and cones.

Recent studies have also demonstrated a light-dependent pigment regeneration pathway mediated by Retinal G protein-coupled opsin (RGR) in RPE (Zhang et al., 2019) and Müller cells (Morshedian et al., 2019). RGR opsin is a non-signaling opsin, expressed in human, bovine and chicken Müller cells as well as in RPE cells (Pandey et al., 1994; Trifunovic et al., 2008; Diaz et al., 2017). Knocking out *Rgr* in the mouse retina results in faster sensitivity loss in cones under constant bright background illumination. Morshedian et al. (2019) found that this light-dependent pigment regeneration is also dependent on the activity of Rdh10 in Müller glia cells. In their model atROL is taken up by Müller cells, and RDH10 and RGR opsin together convert it to 11cROL in the presence of blue light. The alcohol form of 11-*cis* retinoid can then be used by cones but not rods for pigment regeneration. The identity of the RDH enzyme expressed in cones but not in rods catalyzing the oxidation of 11cROL to 11cRAL remains unknown. The ability of cones to maintain sensitivity under conditions of continuous pigment bleaching is expected to be promoted by these light-dependent visual cycles mediated by RGR opsin in both RPE and Müller cells. The contribution of CRALBP- and RGR opsin-mediated as well as non-enzymatic pigment regeneration and canonical visual cycle to the bright light vision and dark adaptation in different species are not known and remains an interesting question to study.

## Concluding Remarks

*Vertebrate* visual and olfactory transduction and *invertebrate* phototransduction rely on prototypical but different GPCR signaling cascades whereas *invertebrate* olfaction uses a more diverse signaling toolbox including fast ionotropic as well as potentially more sensitive metabotropic mechanisms for odorant detection.

Rhabdomeric phototransduction has extremely high amplification and fast response kinetics largely due to Ca^2+^-dependent amplification and feedback mechanisms together with compartmentalization of the transduction into a very small volume (microvilli). This allows invertebrates to detect single photons, and due to the large number of microvilli and bistable visual pigment, they can also sustain vision into bright daylight. Vertebrates developed an alternative strategy to see over a similar range of ambient light. During evolution, the molecular properties of ciliary cone photoreceptors became tuned to generate more sensitive rod phototransduction with high amplification, slower response termination and extremely stable visual pigment to enable single photon detection and vision at night. With a duplex rod/cone retina, vertebrates can also see both at night and in bright sun light in a way that is perhaps more energy efficient compared the invertebrate retina (Fain et al., 2010).

Unlike r-opsins in rhabdomeric photoreceptors, c-opsins in ciliary photoreceptors are not thought to be bistable and several enzymatic pathways to regenerate rod and cone visual pigments exist. The rod pigment is extremely stable with a low rate of spontaneous activation, and once activated it is also very slow to decay back to free opsin and all-*trans*-retinal compared to cone pigments. In addition, cones can use several different visual cycles for faster and more efficient pigment regeneration, enabling vision in very bright light. Most of our information about the function of cones from the past 2–3 decades has been taken from mice, a nocturnal animal with rod-dominant retina. More details about biology of non-human primate and human fovea and para-/perifovea have emerged, but significant gaps in our knowledge remain. For example, the contribution or existence of the different pigment regeneration pathways for bright light vision in the human fovea and macula, specialized to promote high-acuity color vision in bright daylight conditions, are not known. Then again, significant gaps in knowledge about invertebrate phototransduction also remain, where even the identity of second messengers remains controversial.

Although olfactory transduction in vertebrates also uses GPCR signaling cascade to transduce odorant signals, it has much lower amplification compared to phototransduction in either vertebrates or invertebrates. Indeed, the probability for activated olfactory receptor to activate G_olf_ is very low and, whereas rods and rhabdomeric PRs can detect single photons, ~40 odor molecules are required for olfactory sensation in mice. Where vision seems to have evolved in humans to enable high-acuity color vision during the day, mice seem to have retained a significantly larger number of functional olfactory receptors ~1,000, compared to ~400 in humans (Niimura and Nei, 2007).

## Author Contributions

All authors contributed to the writing of this review article to the work and approved it for publication. Figures were generated by FA.

## Conflict of Interest

The authors declare that the research was conducted in the absence of any commercial or financial relationships that could be construed as a potential conflict of interest.
